# Bilateral Conjunctivitis in a Returned Traveller

**DOI:** 10.1371/journal.pntd.0003351

**Published:** 2015-01-15

**Authors:** Sasha R. Fehily, Gail B. Cross, Andrew J. Fuller

**Affiliations:** 1 Infectious Disease Unit, Alfred Hospital, Melbourne, Australia; 2 Department of Microbiology, Alfred Hospital, Melbourne, Australia

## Question

**Figure 1:**
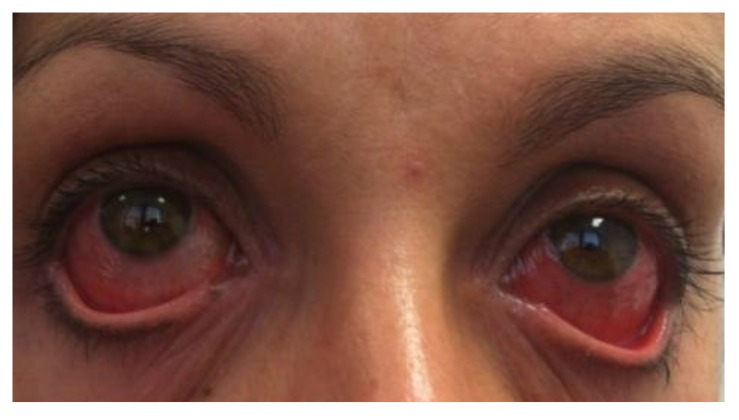
Young woman with significant bilateral conjunctivitis.

A 30-year-old female presented to a tertiary hospital with two weeks of fevers and left upper quadrant abdominal pain after returning from the Gili Islands, Indonesia. She was immunised against hepatitis B, hepatitis A, and typhoid. She did not take malaria prophylaxis and recalls being bitten by insects. Laboratory investigations revealed an elevated C-reactive protein level (158 mg/L), mild thrombocytopenia (148 10^9/L), and deranged liver functions tests. Her malaria smear and blood and urine cultures were negative. Serology for dengue fever, chikungunya, human immunodeficiency virus, hepatitis B, hepatitis C, hepatitis A, leptospirosis, and rickettsia were sent. Two days into the admission, she subsequently developed significant bilateral conjunctivitis and was reviewed by the ophthalmology unit (Fig. 1). Ophthalmoscopy revealed a small, pale, inactive spot on the retina. The lens, macula, and retina otherwise appeared normal. Without antibiotic treatment, the patient’s fevers and abdominal pain resolved, although the conjunctivitis worsened (Fig. 1). The patient in this manuscript has given written informed consent to publication of her case details.

## Diagnosis

### Murine typhus

An immunofluorescence test detecting IgG and IgM antibodies to Rickettsiae (murine typhus group), taken 14 days after the onset of clinical symptoms, was positive, with a titre of 1:8,192. All other serological tests were negative. Repeat Rickettsiae serology performed in convalescence, 14 days after the initial test, demonstrated a rise in the titre to 1:16,384. The patient’s symptoms; a high, rising, positive murine typhus titre; and epidemiological risk factors support the diagnosis of a Rickettsial infection. A 2-fold rise rather than a 4-fold rise in Rickettsiae titre occurred in this case because the first serological sample was taken late in the illness. The patient was treated with oral doxycycline for two weeks and achieved a complete resolution of the conjunctivitis after three weeks.

## Discussion

Murine typhus is endemic to Indonesia, with the prevalence of positive *Rickettsia typhi* antibody levels in humans being one of the highest in the world [1]. Outbreaks have been reported worldwide, but the endemic foci include the Southeast Asian, Mediterranean, and southern United States regions [2]. The environmental circumstances that potentiate the prevalence of this disease are port cities, coastal, and high altitude regions [3, 4]. Infections with this gram-negative intracellular bacterium remain under-diagnosed and underreported, despite being endemic worldwide [4]. Arthropod vectors, commonly the rat flea *Xenopsylla cheopis*, are responsible for the transmission of murine typhus to humans from an animal reservoir [5]. This is consistent with the increased prevalence in tropical port cities, where rats are abundant [3].

In Indonesia, murine typhus has been reported to cause 2.8% of acute undifferentiated fever [3]. The clinical features that manifest after an eight- to 16-day incubation period are usually non-specific, with patients exhibiting fever, headache, and a faint maculopapular rash [3]. However, the complete clinical triad is reported in fewer than 15% of patients [6]. Other clinical features that manifest are arthralgia, myalgia, gastrointestinal symptoms, and acute renal impairment [4]. Acute pulmonary failure and neurological complications are rare, with respective reported prevalence rates of 6%–12% and 15%–45% [2]. Bilateral conjunctivitis has been described in case reports, and a prospective observational study showed that conjunctivitis can occur in up to 21% of cases [2, 4, 7]. As was present in this case, Khairallah et al. observed white lesions on the retina in 50% of cases [8]. Abnormalities in the posterior segment of the eye, including fundal lesions and chorioretinal changes, were identified in 88.9% of the patients studied [8].

Elevated aminotransferases, hypoalbuminaemia, and hyponatremia are the biochemical abnormalities frequently reported [2]. Haematological findings that occur include leucocytosis, leukopenia, anaemia, and thrombocytopenia [2]. Although the infection is usually clinically mild, it can result in a severe illness and may even be fatal in up to 4% of hospitalised patients that are not treated with antibiotics [9].

The definitive diagnosis is based on epidemiological data, patient history, clinical signs, and positive convalescent serological testing [8]. This is notoriously difficult and frequently delayed given the non-specific clinical features. Additionally, there are no adequate diagnostic tests during the acute phase of illness. The gold standard for diagnosis is a 4-fold rise on the immunofluorescence assay detecting *R. typhi* IgM and IgG antibodies [7]. The preferred treatment of murine typhus is early initiation of antimicrobial therapy, with the first-line agent being doxycycline [8].

**Key Learning Points**

- Murine typhus is an under-diagnosed infectious disease in returned travelers.
- Arthropod vectors are responsible for the transmission of this gram-negative intracellular bacterium to humans.
- Murine typhus should be considered in the differential diagnosis of returned travellers with bilateral conjunctivitis and fevers (Table 1, Table 2).
- There are no satisfactory diagnostic tests during the acute phase of illness.
- Doxycycline is considered to be the first-line antimicrobial therapy for the treatment of murine typhus.

**Table 1:** Infectious causes of conjunctivitis—bacterial.

**Diseases**	**Organism (Vector)**	**Vector (type)**	**Unilateral or bilateral conjunctivitis**	**Ocular manifestations and other common features**
Bacterial Conjunctivitis [10]	*Staphylococcus aureus, Streptococcus pneumonia, Haemophilus influenza, Moraxella catarrhalis*	Nil	Unilateral	Conjunctivitis with mucopurulent discharge, conjunctival hyperemia.
Hyperacute Bacterial Conjunctivitis [10]	*Neisseria Gonorrhoea*	Nil	Unilateral or bilateral	Conjunctivitis with severe copious purulent discharge, eyelid swelling, lymphadenopathy.
Adult Inclusion Conjunctivitis [10]	*Chlamydia trachomatis*	Nil	Unilateral or bilateral	Conjunctivitis with mucopurulent discharge, corneal scarring, blindness.
Rickettsioses [11]	Spotted fever group, Typhus group, Scrub typhus group	Ticks, fleas, mites, lice	Unilateral or bilateral	Systemic febrile illness, conjunctivitis, inner retinitis, headache, malaise, rash.
Leptospirosis [12]	*Leptospires*	Nil	Unilateral or bilateral	Systemic febrile illness, subconjunctival hemorrhage, conjunctivitis, uveitis, headache, arthralgia, rash, gastrointestinal symptoms.

**Table 2:** Infectious causes of conjunctivitis—viral.

**Diseases**	**Organism (Vector)**	**Vector (type)**	**Unilateral or bilateral conjunctivitis**	**Ocular manifestations and other common features**
Viral Conjunctivitis [10]	65% due to *Adenovirus* strains	Nil	Unilateral or bilateral	Pharyngoconjunctival fever: pharyngitis, conjunctivitis, high fever, bilateral lymphadenopathy. Epidemic keratoconjunctivitis: conjunctivitis with watery discharge, hyperemia, chemosis, ipsilateral lymphadenopathy.
Herpes Zoster virus [10]	*Herpesvirus*	Nil	Unilateral	Conjunctivitis with watery discharge, vesicular eyelid lesions, corneal ulceration.
Herpes Simplex virus [10]	*Herpesvirus*	Nil	Unilateral	Conjunctivitis with watery discharge, vesicular eyelid lesions.
West Nile virus [11, 13]	*Flavivirus*	Mosquitoes (*Culex*)	Bilateral	Systemic febrile illness, multifocal chorioretinitis, myalgia, arthralgia, rash, gastrointestinal symptoms.
Dengue fever [11]	*Flavivirus*	Mosquitoes (*Aedes aegypti*)	Bilateral	Systemic febrile illness, anterior uveitis, subconjunctival haemorrhage, scotoma, headache, myalgia, rash.
Chikungunya [11, 14]	*Alphavirus*	Mosquitoes (*Aedes aegypti*)	Unilateral or bilateral	Systemic febrile illness, anterior uveitis, retinitis, headache, rash, epistaxis, oedema.
Zika Virus [15]	*Flavivirus*	Nil	Bilateral	Systemic febrile illness, conjunctivitis, arthralgia, rash.
Rift Valley fever [11, 16]	*Bunyaviridae*	Ticks, Mosquitoes, Sand flies	Unilateral or bilateral	Systemic febrile illness, macular retinitis, headache, myalgia, arthralgia, gastrointestinal symptoms.
Measles [17]	*Measles virus*	Nil	Bilateral	Systemic febrile illness, conjunctivitis, cough, rhinitis, rash.
Rubella	*Rubella virus*	Nil	Bilateral	Mild fever, conjunctivitis, headache, myalgia, rash.
H1N1 [11, 18, 19]	*Influenza A H1N1*	Nil	Bilateral	Systemic febrile illness, cough, myalgia. Conjunctivitis, uveitis and retinitis are infrequently reported.

## References

[1] Richards AL, Soeatmadji DW, Widodo MA, Sardjono TW, Yanuwiadi B, et al (1997) Seroepidemiological evidence for murine and scrub typhus in Malang, Indonesia. Am J Trop Med Hyg 57: 91–5. 9242326 10.4269/ajtmh.1997.57.91

[2] Chaliotis G, Kritsotakis EI, Psaroulaki A, Tselentis Y, Gikas A (2012) Murine typhus in central Greece: epidemiological, clinical, laboratory, and therapeutic-response features of 90 cases. Int J Infect Dis 16: e591–e596. 10.1016/j.ijid.2012.03.010 22658872

[3] Gasem MH, Wagenaar JFP, Goris MGA, Adi MS, Isbandrio BB, et al (2009) Murine typhus and leptospirosis as causes of acute undifferentiated fever, indonesia. Emerg Infect Dis 15: 975–977. 10.3201/eid1506.081405 19523308 PMC2727336

[4] Parola P, Vogelaers D, Roure C, Janbon F, Raoult D (1998) Murine typhus in travelers returning from Indonesia. Emerg Infect Dis 4: 677–680. 10.3201/eid0404.980423 9866749 PMC2640266

[5] Richards AL, Rahardjo E, Rusjdi AF, Kelly AJ, Dasch GA, et al (2002) Evidence of rickettsia typhi and the potential for murine typhus in jayapura, irian jaya, indonesia. Am J Trop Med 66: 431–434. 10.4269/ajtmh.2002.66.431 12164301

[6] Dumler JS, Taylor JP, Walker DH (1991) Clinical and laboratory features of murine typhus in Texas, 1980 through 1987. JAMA 266: 1365–70. 10.1001/jama.266.10.1365 1880866

[7] Stockdale AJ, Weekes MP, Keily B, Lever AM (2011) Severe typhus group rickettsiosis complicated by pulmonary edema in a returning traveler from Indonesia. Am J Trop Med Hyg 85: 1121–1123. 10.4269/ajtmh.2011.11-0340 22144455 PMC3225163

[8] Khairallah M, Yahia SB, Toumi A, Jelliti B, Loussaief C, et al (2009) Ocular manifestations associated with murine typhus. Br J Opthalmol 93: 938–942. 10.1136/bjo.2008.156059 19414440

[9] Civen R, Ngo V (2008) Murine typhus: an unrecognized suburban vectorborne disease. Clin Infect Dis 46: 913–918. 10.1086/527443 18260783

[10] Azari A, Barney NP (2013) Conjunctivitis. JAMA 310(16): 1721 10.1001/jama.2013.280318 24150468 PMC4049531

[11] Khairallah M, Kahloun R, Ben Yahia S, et al (2013) New infectious etiologies for posterior uveitis. Ophthalmic Res; 49:66–72. 10.1159/000344009 23258387

[12] Rathinam SR (2005) Ocular manifestations of leptospirosis. J Postgrad Med. 51(3):189–94. 16333191

[13] Khairallah M, Ben Yahia S, Ladjimi A, et al (2004) Chorioretinal involvement in patients with West Nile virus infection. Ophthalmology 111:2065–2070. 10.1016/j.ophtha.2004.03.032 15522373

[14] Mahendradas P, Avadhani K, Shetty R (2013). Chikungunya and the eye: a review. J Ophthalmic Inflamm Infect; 3:35 10.1186/1869-5760-3-35 23514031 PMC3605073

[15] Hayes EB (2009) Zika virus outside Africa. Emerg Infect Dis 15(9): 1347–1350. 10.3201/eid1509.090442 19788800 PMC2819875

[16] Al-Hazmi A, Al-Rajhi AA, Abboud EB, et al (2005) Ocular complications of Rift Valley fever outbreak in Saudi Arabia. Ophthalmology; 112:313–318. 10.1016/j.ophtha.2004.09.018 15691569

[17] Sabella C. (2010) Measles: not just a childhood rash. *Cleve Clin J Med.* Mar 77(3):207–13. 10.3949/ccjm.77a.09123 20200172

[18] Rifkin L, Schaal S. (2012) H1N1-associated acute retinitis. Ocul Immunol Inflamm; 20:230–232. 10.3109/09273948.2012.674611 22537287

[19] Mansour DE, El-Shazly AA, Elawamry AI, Ismail AT. (2012) Comparison of ocular findings in patients with H1N1 influenza infection versus patients receiving influenza vaccine during a pandemic. Ophthalmic Res; 48:134–138. 10.1159/000337138 22572924

